# IsomiR Utility in ALS Prognostication

**DOI:** 10.1101/2025.05.12.25325848

**Published:** 2025-05-14

**Authors:** Y. Cohen, I. Sinai, I. Magen, M. Y. Danino, J. Wuu, A. Malaspina, M. Benatar, E. Hornstein

## Abstract

Amyotrophic lateral sclerosis (ALS) is a fatal neurodegenerative disease characterized by progressive motor neuron loss. IsomiRs are microRNA isoforms that arise from alternative processing or editing events during miRNA biogenesis. While isomiRs may carry distinct biological and clinical relevance, their potential as cell-free biomarkers in neurodegeneration remains largely unexplored. Intriguingly, loss of TAR DNA-binding protein 43 (TDP-43) nuclear function is a hallmark of disease and is known to impair isomiR expression. Here, we investigated the prognostic utility of plasma isomiRs in ALS, using next-generation sequencing. We profiled cell-free isomiRs in 154 ALS patients from a British cohort and identified higher levels of one isomiR, let-7g-5p.t, to be associated with longer survival. This finding was independently validated in an international ALS cohort of 200 patients and was in two orthogonal approaches. let-7g-5p.t prognostic utility was comparable to that of neurofilament light chain (NfL) or miR-181. These results establish isomiRs as a novel class of blood-based biomarkers in ALS with potential to refine prognostication in clinical trials for neurodegenerative diseases.

## Full Text Availability

The license terms selected by the author(s) for this preprint version do not permit archiving in PMC. The full text is available from the preprint server.

