# Supplementary material for: IsomiR Utility in Amyotrophic Lateral Sclerosis Prognostication
Source: medRxiv. 2025 Sep 15:2025.05.12.25325848. Preprint. [Version 4] doi: 10.1101/2025.05.12.25325848 (PMC12132153; doi:10.1101/2025.05.12.25325848)
Supplement: Supplementary file 1 [file media-1.pdf]

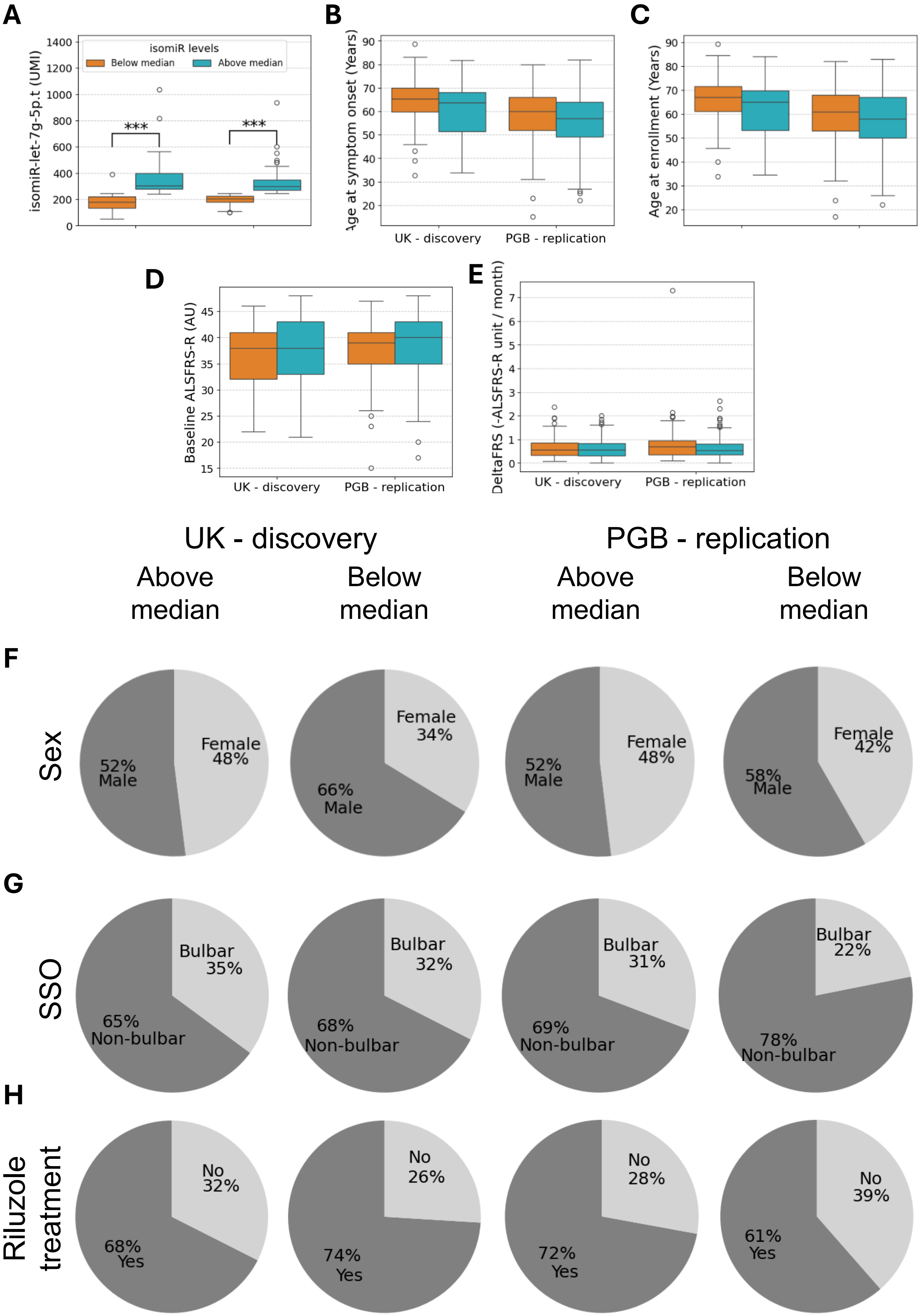

A
isomiR levels
Below median
Above median
isomiR-let-7g-5p.t (UMI)
***
***
B
Age at symptom onset (Years)
UK - discovery
PGB - replication
C
Age at enrollment (Years)
D
Baseline ALSFRS-R (AU)
UK - discovery
PGB - replication
E
DeltaFRS (-ALSFRS-R unit / month)
UK - discovery
PGB - replication
UK - discovery
PGB - replication
Above median
Below median
Above median
Below median
F
Sex
Female 48%
52% Male
Female 34%
66% Male
Female 48%
52% Male
Female 42%
58% Male
G
SSO
Bulbar 35%
65% Non-bulbar
Bulbar 32%
68% Non-bulbar
Bulbar 31%
69% Non-bulbar
Bulbar 22%
78% Non-bulbar
H
Riluzole treatment
No 32%
68% Yes
No 26%
74% Yes
No 28%
72% Yes
No 39%
61% Yes
