# Supplementary material for: IsomiR Utility in Amyotrophic Lateral Sclerosis Prognostication
Source: medRxiv. 2025 Sep 15:2025.05.12.25325848. Preprint. [Version 4] doi: 10.1101/2025.05.12.25325848 (PMC12132153; doi:10.1101/2025.05.12.25325848)
Supplement: Supplementary file 2 [file media-2.pdf]

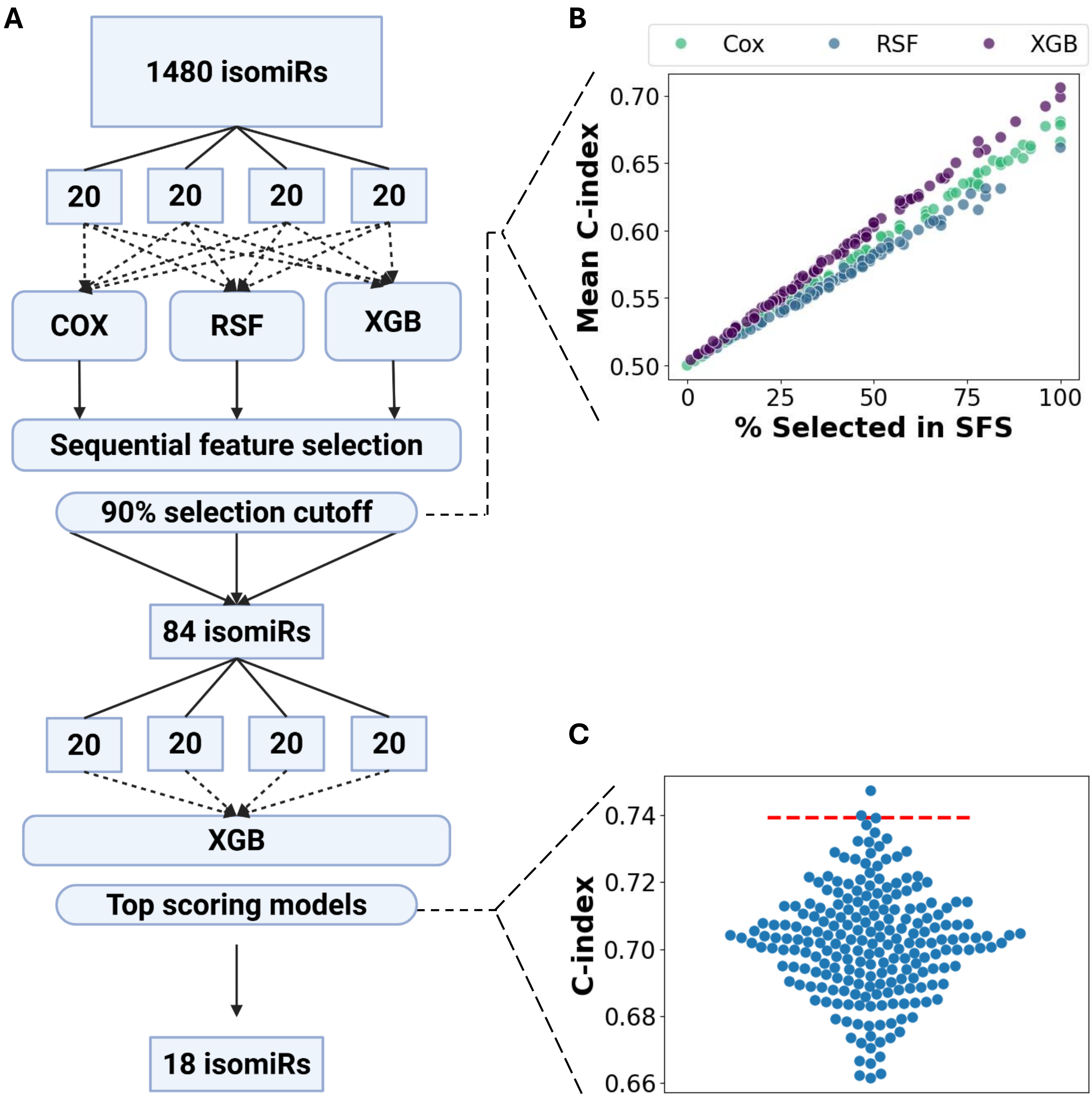

A
1480 isomiRs
20
20
20
20
COX
RSF
XGB
Sequential feature selection
90% selection cutoff
84 isomiRs
20
20
20
20
XGB
Top scoring models
18 isomiRs
B
Cox
RSF
XGB
Mean C-index
0.70
0.65
0.60
0.55
0.50
0
25
50
75
100
% Selected in SFS
C
C-index
0.74
0.72
0.70
0.68
0.66
