# Supplementary material for: IsomiR Utility in Amyotrophic Lateral Sclerosis Prognostication
Source: medRxiv. 2025 Sep 15:2025.05.12.25325848. Preprint. [Version 4] doi: 10.1101/2025.05.12.25325848 (PMC12132153; doi:10.1101/2025.05.12.25325848)
Supplement: Supplementary file 3 [file media-3.pdf]

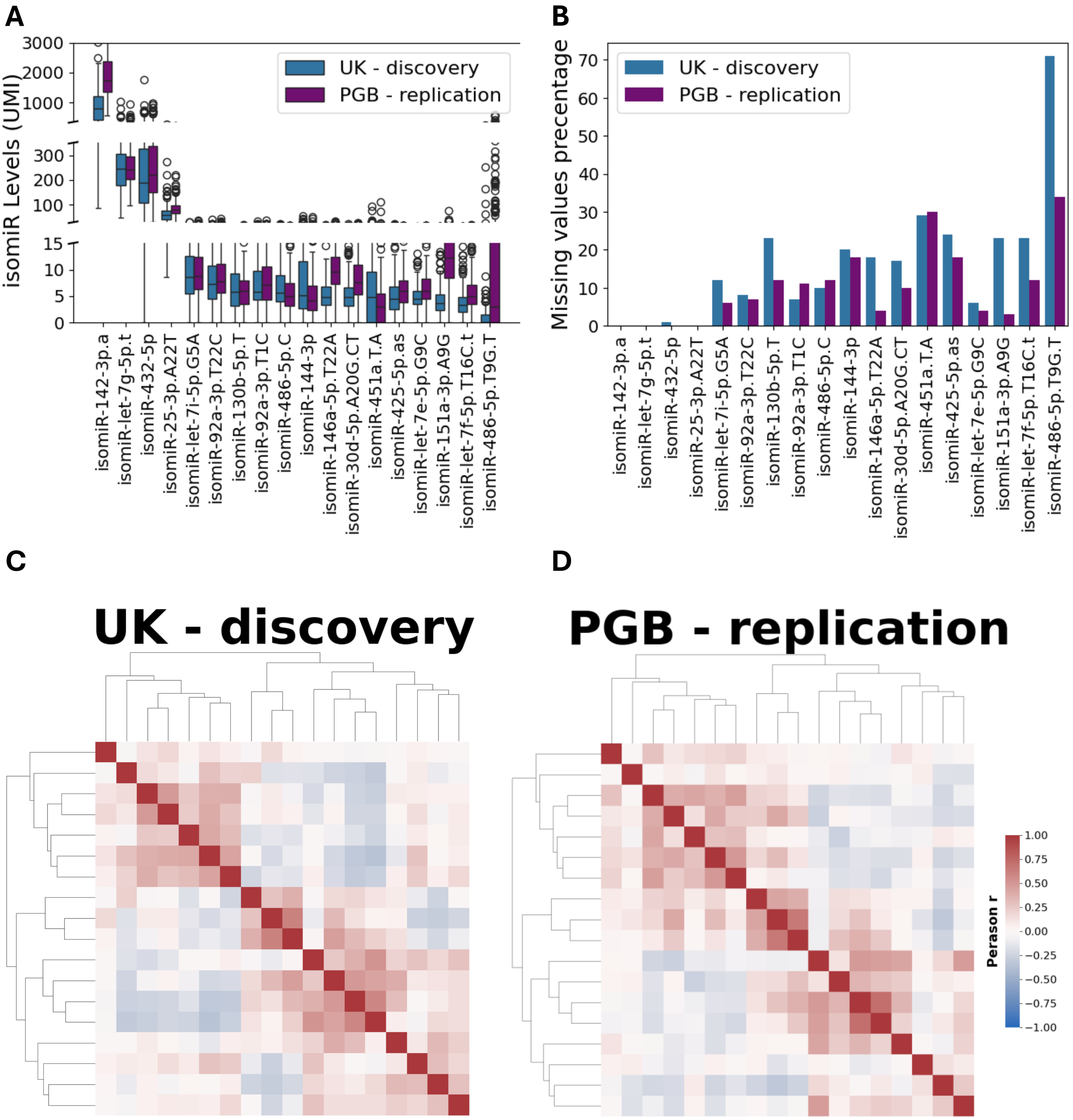
A
B
C
D
UK - discovery
PGB - replication
isomiR Levels (UMI)
Missing values precentage
isomiR-142-3p.a
isomiR-let-7g-5p.t
isomiR-432-5p
isomiR-25-3p.A22T
isomiR-let-7i-5p.G5A
isomiR-92a-3p.T22C
isomiR-130b-5p.T
isomiR-92a-3p.T1C
isomiR-486-5p.C
isomiR-144-3p
isomiR-146a-5p.T22A
isomiR-30d-5p.A20G.CT
isomiR-451a.T.A
isomiR-425-5p.as
isomiR-let-7e-5p.G9C
isomiR-151a-3p.A9G
isomiR-let-7f-5p.T16C.t
isomiR-486-5p.T9G.T
UK - discovery
PGB - replication
Perason r
