# Supplementary material for: IsomiR Utility in Amyotrophic Lateral Sclerosis Prognostication
Source: medRxiv. 2025 Sep 15:2025.05.12.25325848. Preprint. [Version 4] doi: 10.1101/2025.05.12.25325848 (PMC12132153; doi:10.1101/2025.05.12.25325848)
Supplement: Supplementary file 4 [file media-4.pdf]

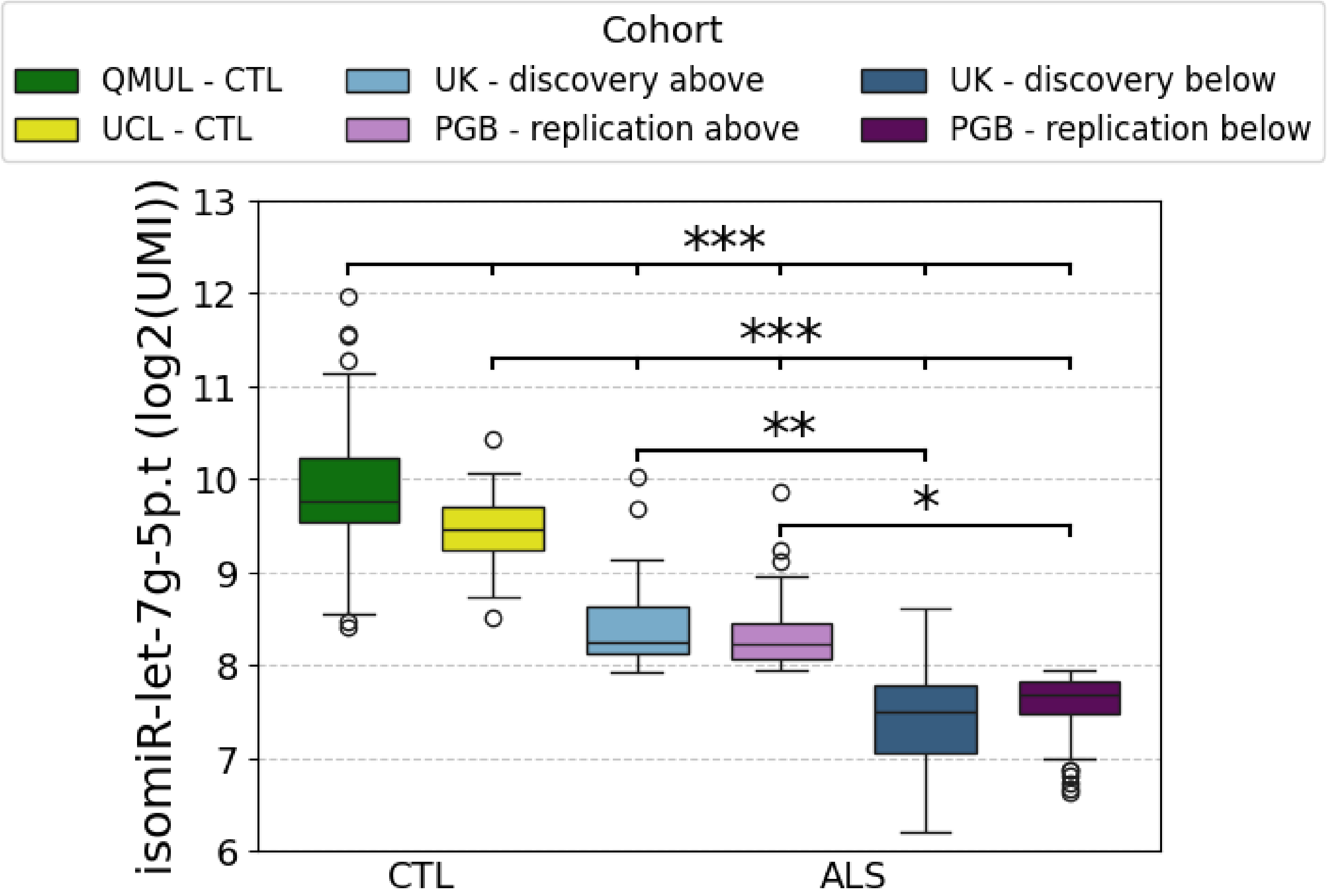
Cohort
QMUL - CTL
UCL - CTL
UK - discovery above
PGB - replication above
UK - discovery below
PGB - replication below
isomiR-let-7g-5p.t (log2(UMI))
13
12
11
10
9
8
7
6
***
***
**
*
CTL
ALS
