# Supplementary material for: IsomiR Utility in Amyotrophic Lateral Sclerosis Prognostication
Source: medRxiv. 2025 Sep 15:2025.05.12.25325848. Preprint. [Version 4] doi: 10.1101/2025.05.12.25325848 (PMC12132153; doi:10.1101/2025.05.12.25325848)
Supplement: Supplementary file 5 [file media-5.pdf]

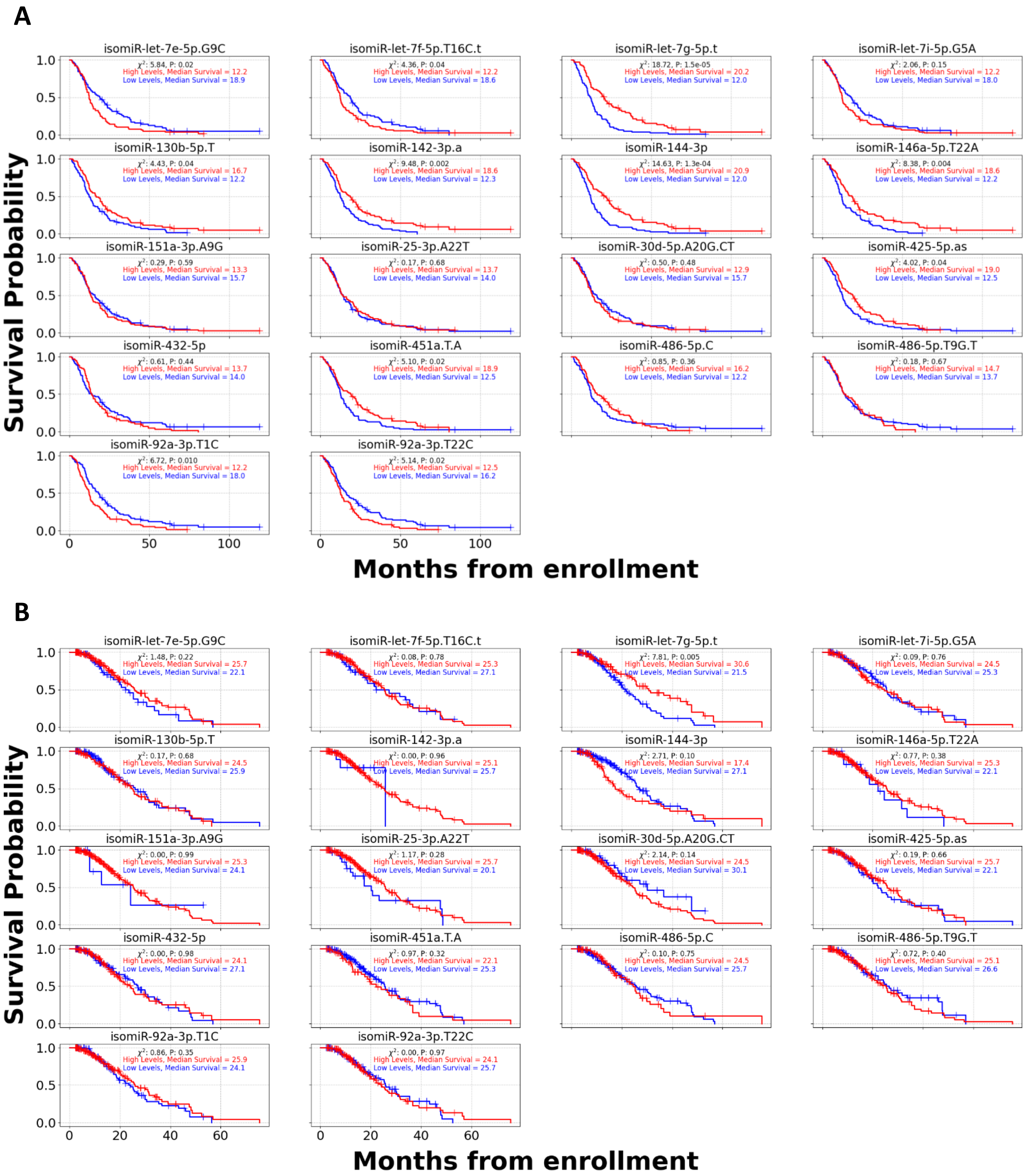
A
Survival Probability
Months from enrollment
isomiR-let-7e-5p.G9C
χ^2: 5.84, P: 0.02
High Levels, Median Survival = 12.2
Low Levels, Median Survival = 18.9
isomiR-let-7f-5p.T16C.t
χ^2: 4.36, P: 0.04
High Levels, Median Survival = 12.2
Low Levels, Median Survival = 18.6
isomiR-let-7g-5p.t
χ^2: 18.72, P: 1.5e-05
High Levels, Median Survival = 20.2
Low Levels, Median Survival = 12.0
isomiR-let-7i-5p.G5A
χ^2: 2.06, P: 0.15
High Levels, Median Survival = 12.2
Low Levels, Median Survival = 18.0
isomiR-130b-5p.T
χ^2: 4.43, P: 0.04
High Levels, Median Survival = 16.7
Low Levels, Median Survival = 12.2
isomiR-142-3p.a
χ^2: 9.48, P: 0.002
High Levels, Median Survival = 18.6
Low Levels, Median Survival = 12.3
isomiR-144-3p
χ^2: 14.63, P: 1.3e-04
High Levels, Median Survival = 20.9
Low Levels, Median Survival = 12.0
isomiR-146a-5p.T22A
χ^2: 8.38, P: 0.004
High Levels, Median Survival = 18.6
Low Levels, Median Survival = 12.2
isomiR-151a-3p.A9G
χ^2: 0.29, P: 0.59
High Levels, Median Survival = 13.3
Low Levels, Median Survival = 15.7
isomiR-25-3p.A22T
χ^2: 0.17, P: 0.68
High Levels, Median Survival = 13.7
Low Levels, Median Survival = 14.0
isomiR-30d-5p.A20G.CT
χ^2: 0.50, P: 0.48
High Levels, Median Survival = 12.9
Low Levels, Median Survival = 15.7
isomiR-425-5p.as
χ^2: 4.02, P: 0.04
High Levels, Median Survival = 19.0
Low Levels, Median Survival = 12.5
isomiR-432-5p
χ^2: 0.61, P: 0.44
High Levels, Median Survival = 13.7
Low Levels, Median Survival = 14.0
isomiR-451a.T.A
χ^2: 5.10, P: 0.02
High Levels, Median Survival = 18.9
Low Levels, Median Survival = 12.5
isomiR-486-5p.C
χ^2: 0.85, P: 0.36
High Levels, Median Survival = 16.2
Low Levels, Median Survival = 12.2
isomiR-486-5p.T9G.T
χ^2: 0.18, P: 0.67
High Levels, Median Survival = 14.7
Low Levels, Median Survival = 13.7
isomiR-92a-3p.T1C
χ^2: 6.72, P: 0.010
High Levels, Median Survival = 12.2
Low Levels, Median Survival = 18.0
isomiR-92a-3p.T22C
χ^2: 5.14, P: 0.02
High Levels, Median Survival = 12.5
Low Levels, Median Survival = 16.2
B
Survival Probability
Months from enrollment
isomiR-let-7e-5p.G9C
χ^2: 1.48, P: 0.22
High Levels, Median Survival = 25.7
Low Levels, Median Survival = 22.1
isomiR-let-7f-5p.T16C.t
χ^2: 0.08, P: 0.78
High Levels, Median Survival = 25.3
Low Levels, Median Survival = 27.1
isomiR-let-7g-5p.t
χ^2: 7.81, P: 0.005
High Levels, Median Survival = 30.6
Low Levels, Median Survival = 21.5
isomiR-let-7i-5p.G5A
χ^2: 0.09, P: 0.76
High Levels, Median Survival = 24.5
Low Levels, Median Survival = 25.3
isomiR-130b-5p.T
χ^2: 0.17, P: 0.68
High Levels, Median Survival = 24.5
Low Levels, Median Survival = 25.9
isomiR-142-3p.a
χ^2: 0.00, P: 0.96
High Levels, Median Survival = 25.1
Low Levels, Median Survival = 25.7
isomiR-144-3p
χ^2: 2.71, P: 0.10
High Levels, Median Survival = 17.4
Low Levels, Median Survival = 27.1
isomiR-146a-5p.T22A
χ^2: 0.77, P: 0.38
High Levels, Median Survival = 25.3
Low Levels, Median Survival = 22.1
isomiR-151a-3p.A9G
χ^2: 0.00, P: 0.99
High Levels, Median Survival = 25.3
Low Levels, Median Survival = 24.1
isomiR-25-3p.A22T
χ^2: 1.17, P: 0.28
High Levels, Median Survival = 25.7
Low Levels, Median Survival = 20.1
isomiR-30d-5p.A20G.CT
χ^2: 2.14, P: 0.14
High Levels, Median Survival = 24.5
Low Levels, Median Survival = 30.1
isomiR-425-5p.as
χ^2: 0.19, P: 0.66
High Levels, Median Survival = 25.7
Low Levels, Median Survival = 22.1
isomiR-432-5p
χ^2: 0.00, P: 0.98
High Levels, Median Survival = 24.1
Low Levels, Median Survival = 27.1
isomiR-451a.T.A
χ^2: 0.97, P: 0.32
High Levels, Median Survival = 22.1
Low Levels, Median Survival = 25.3
isomiR-486-5p.C
χ^2: 0.10, P: 0.75
High Levels, Median Survival = 24.5
Low Levels, Median Survival = 25.7
isomiR-486-5p.T9G.T
χ^2: 0.72, P: 0.40
High Levels, Median Survival = 25.1
Low Levels, Median Survival = 26.6
isomiR-92a-3p.T1C
χ^2: 0.86, P: 0.35
High Levels, Median Survival = 25.9
Low Levels, Median Survival = 24.1
isomiR-92a-3p.T22C
χ^2: 0.00, P: 0.97
High Levels, Median Survival = 24.1
Low Levels, Median Survival = 25.7
