# Supplementary material for: IsomiR Utility in Amyotrophic Lateral Sclerosis Prognostication
Source: medRxiv. 2025 Sep 15:2025.05.12.25325848. Preprint. [Version 4] doi: 10.1101/2025.05.12.25325848 (PMC12132153; doi:10.1101/2025.05.12.25325848)
Supplement: Supplementary file 6 [file media-6.pdf]

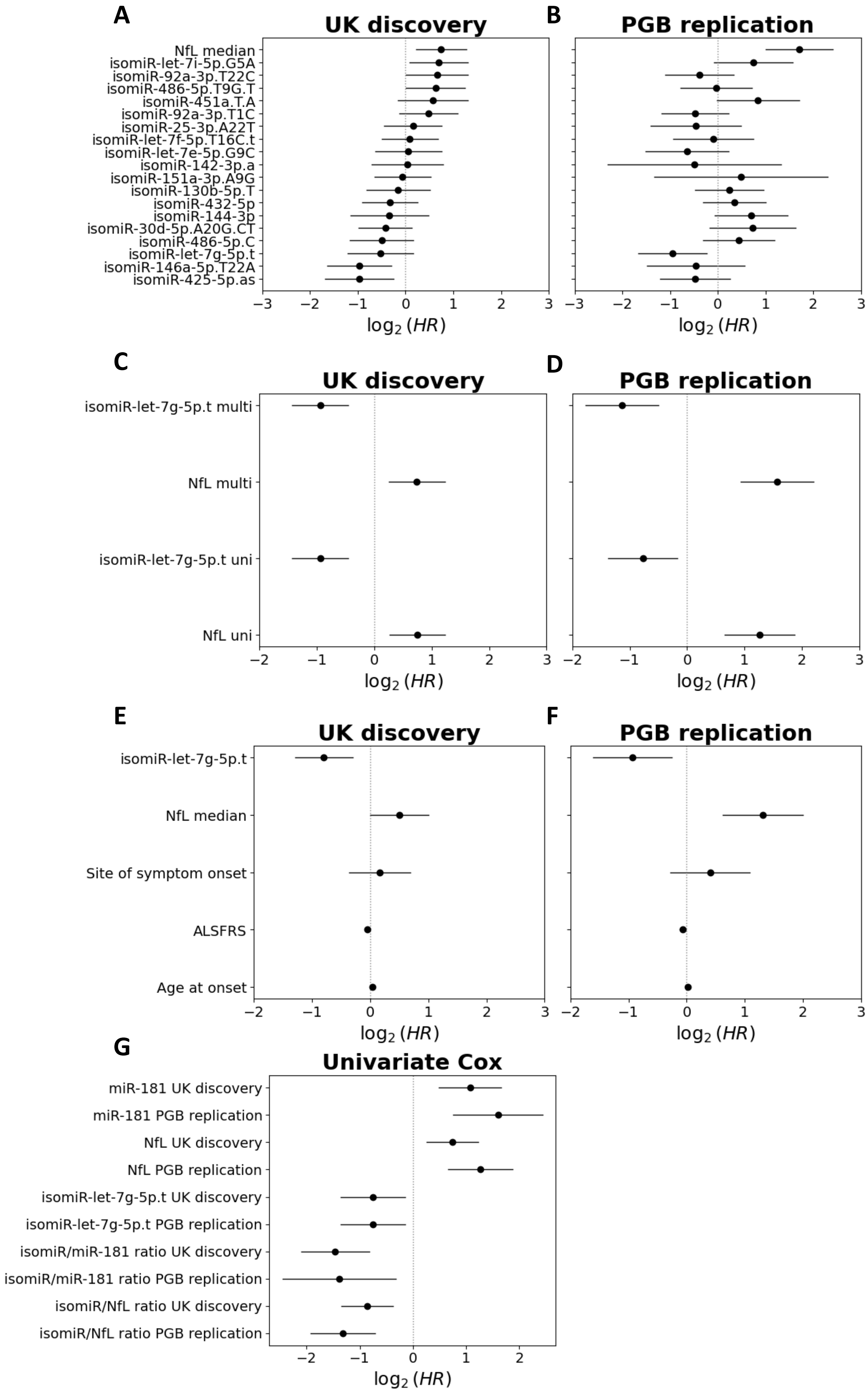

A
UK discovery
NfL median
isomiR-let-7i-5p.G5A
isomiR-92a-3p.T22C
isomiR-486-5p.T9G.T
isomiR-451a.T.A
isomiR-92a-3p.T1C
isomiR-25-3p.A22T
isomiR-let-7f-5p.T16C.t
isomiR-let-7e-5p.G9C
isomiR-142-3p.a
isomiR-151a-3p.A9G
isomiR-130b-5p.T
isomiR-432-5p
isomiR-144-3p
isomiR-30d-5p.A20G.CT
isomiR-486-5p.C
isomiR-let-7g-5p.t
isomiR-146a-5p.T22A
isomiR-425-5p.as
$\log_2(HR)$
B
PGB replication
$\log_2(HR)$
C
UK discovery
isomiR-let-7g-5p.t multi
NfL multi
isomiR-let-7g-5p.t uni
NfL uni
$\log_2(HR)$
D
PGB replication
$\log_2(HR)$
E
UK discovery
isomiR-let-7g-5p.t
NfL median
Site of symptom onset
ALSFRS
Age at onset
$\log_2(HR)$
F
PGB replication
$\log_2(HR)$
G
Univariate Cox
miR-181 UK discovery
miR-181 PGB replication
NfL UK discovery
NfL PGB replication
isomiR-let-7g-5p.t UK discovery
isomiR-let-7g-5p.t PGB replication
isomiR/miR-181 ratio UK discovery
isomiR/miR-181 ratio PGB replication
isomiR/NfL ratio UK discovery
isomiR/NfL ratio PGB replication
$\log_2(HR)$
